# Role of NeuroD1 on the negative regulation of *Pomc* expression by glucocorticoid

**DOI:** 10.1371/journal.pone.0175435

**Published:** 2017-04-13

**Authors:** Rehana Parvin, Akiko Saito-Hakoda, Hiroki Shimada, Kyoko Shimizu, Erika Noro, Yasumasa Iwasaki, Ken Fujiwara, Atsushi Yokoyama, Akira Sugawara

**Affiliations:** 1 Department of Molecular Endocrinology, Tohoku University Graduate School of Medicine, Sendai, Miyagi, Japan; 2 Health Service Center, Kochi University, Kochi, Kochi, Japan; 3 Division of Histology and Cell Biology, Department of Anatomy, Jichi Medical University School of Medicine, Tochigi, Japan; Harvard Medical School, UNITED STATES

## Abstract

The mechanism of the negative regulation of proopiomelanocortin gene (*Pomc*) by glucocorticoids (Gcs) is still unclear in many points. Here, we demonstrated the involvement of neurogenic differentiation factor 1 (NeuroD1) in the Gc-mediated negative regulation of *Pomc*. Murine pituitary adrenocorticotropic hormone (ACTH) producing corticotroph tumor-derived AtT20 cells were treated with dexamethasone (DEX) (1–100 nM) and cultured for 24 hrs. Thereafter, *Pomc* mRNA expression was studied by quantitative real-time PCR and rat *Pomc* promoter (-703/+58) activity was examined by luciferase assay. Both *Pomc* mRNA expression and *Pomc* promoter activity were inhibited by DEX in a dose-dependent manner. Deletion and point mutant analyses of *Pomc* promoter suggested that the DEX-mediated transcriptional repression was mediated via E-box that exists at -376/-371 in the promoter. Since NeuroD1 is known to bind to and activate E-box of the *Pomc* promoter, we next examined the effect of DEX on NeuroD1 expression. Interestingly, DEX dose-dependently inhibited *NeuroD1* mRNA expression, mouse *NeuroD1* promoter (-2.2-kb) activity, and NeuroD1 protein expression in AtT20 cells. In addition, we confirmed the inhibitory effect of DEX on the interaction of NeuroD1 and E-box on *Pomc* promoter by chromatin immunoprecipitation (ChIP) assay. Finally, overexpression of mouse NeuroD1 could rescue the DEX-mediated inhibition of *Pomc* mRNA expression and *Pomc* promoter activity. Taken together, it is suggested that the suppression of *NeuroD1* expression and the inhibition of NeuroD1/E-box interaction may play an important role in the Gc-mediated negative regulation of *Pomc*.

## Introduction

Class II basic helix-loop-helix (bHLH) factor, neurogenic differentiation factor 1 (NeuroD1) or Beta2 (β2) is characterized by its tissue specific expression [1]. NeuroD1 is expressed in progenitor cells and differentiated endocrine cells of the pancreas [2,3] and in the neuroectoderm [4]. NeuroD1 also plays an important role in the differentiation, morphogenesis and maintenance of the (central) nervous system [5,6]. Corticotroph cells of the anterior pituitary, which produce multifaceted protein proopiomelanocortin (POMC), express mainly NeuroD1 [7], which promotes the expression of POMC gene (*Pomc*). NeuroD1 forms a heterodimer with E proteins (e.g., E47/Pan1), which are a class I bHLH factor. The NeuroD1/Pan1 heterodimer binds to the E-box on *Pomc* promoter then activates *Pomc* transcription [7]. The bHLH heterodimer acts together with pituitary T-box transcription factor Tbx19, which is dependent on another transcription factor, Ptx [8]. When NeuroD1 is required for DNA sequence recognition of the E-box [1,8], the Pan1 part of the bHLH heterodimer interacts with the Ptx1 [7,9] and Tpit [8].

The *Pomc* is expressed significantly in a number of mammalian tissues including the anterior and intermediate pituitary, the immune system, skin and hypothalamus [10,11,12]. In these tissues, POMC is cleaved into a variety of smaller peptides including ACTH, β-endorphin and α-, β- and γ-melanocyte-stimulating hormones (MSH). The repertoire of products derived from POMC by any tissue is determined by the specificities of the convertases expressed in the secretory pathway [13,14]. Prohormone convertase 1 (PC1) is expressed in corticotrophs of the anterior pituitary and in melanotrophs of the intermediate lobe of the pituitary; whereas prohormone convertase 2 (PC2) is expressed in melanotrophs of the intermediate lobe of the pituitary and arcuate nuclei of the hypothalamus. PC1 cleaves POMC to ACTH, while PC2 cleaves ACTH further to yield α-MSH. Thus, secretion of ACTH is the principal controller of adrenal steroidogenesis from the anterior pituitary [15]. ACTH and α-MSH are products of post translational splicing of a precursor molecule, POMC. The corticotrophs secrete mainly ACTH, whereas the melanotrophs mainly α-MSH.

The regulation of *Pomc* is also tissue specific [16]. Although adrenal glucocorticoids (Gcs) upregulate *Pomc* expression in the hypothalamus [17], they negatively regulate *Pomc* transcription and ACTH secretion in pituitary corticotroph cells [18,19,20]. In general, Gcs show their biological activities by binding to a glucocorticoid receptor (GR) [21]. GR resides in the cytoplasm before the presence of Gcs [22]. When Gcs bind to GR, GR translocates to the nucleus [23]. Gcs bound GRs form as a homodimer that binds to Gc-response element (GRE), then activates target gene transcription with transcription machinery [24]. The GRs homodimer also binds to negative GRE (nGRE) of the *Pomc* promoter, and this nGRE complex represses *Pomc* transcription in corticotrophs [25].

It remains to be elucidated whether other transcription factors are involved in the Gc-mediated repression of *Pomc* transcription in pituitary corticotroph cells. In addition to NeuroD1 [7], Nur77, Nurr1, Tpit and Pitx are known to activate *Pomc* transcription [26,27]. Among them, Nur77 [26] and Tpit/PitxRE [27] have been demonstrated to be involved in the Gc-mediated *Pomc* repression. However, the function of NeuroD1 in the negative regulation of *Pomc* has not yet been demonstrated. In this study, we have attempted to examine the involvement of NeuroD1 in the dexamethasone (DEX)-mediated repression of *Pomc* transcription.

## Materials and methods

### Reagents

DEX, a synthetic Gc, was purchased from Sigma-Aldrich (St. Louis, MO). DEX was dissolved in 100% ethanol at 1 mM and stored at -20°C. These stocks were diluted with 100% ethanol to the desired concentration immediately before each experiment and maintained at a final ethanol concentration of at 0.1%.

### Plasmids

Subcloned chimeric constructs containing the rat *Pomc* genomic DNA and luciferase cDNA (pGL3-Basic, Promega, Madison, WI) were used for the transient transfection studies: r*Pomc*-Luc (-703/+58-Luc: harboring the rat *Pomc* 5’-flanking region from -703 to +58 relative to the transcription start site upstream of the luciferase cDNA in pGL3-Basic), -429/+58-Luc; -379/+58-Luc, and -359/+58-luc, and E-box (NeuroD1 binding element) mutant in r*Pomc*-Luc (r*Pomc*-Luc-Ebox_Neuro_-Mut) were described in our previous papers [28,29]. β-galactosidase control plasmid in pRSV (pRSV-β-gal) and in pCMV (pCMV-β-gal) were purchased from Clontech (Mountain View, CA). Beta2 (NeuroD1) overexpression plasmid was a gift from Dr. Debra E. Bramblett (Texas Tech University Health Sciences Center, El Paso, TX). pcDNA3 expression plasmid was purchased from Invitrogen (Carsbad, CA). The full-length mouse *NeuroD1* promoter construct m*NeuroD1*-Luc (-2.2-kb/+150-Luc: harboring the mouse *NeuroD1* 5’-flanking region from -2.2-kb to +150 relative to the transcription start site upstream of the luciferase cDNA in pGL3-Basic, also termed ND full) and *NeuroD1* promoter deletion constructs termed ND mut1, mut2, mut3, and mut4 in the reporter construct pGL3-Basic [30] were kind gifts from Dr Lori Sussel (Columbia University, New York, NY).

### Cell culture

AtT20 cells were grown with Dulbecco’s modified Eagle medium (DMEM) supplemented with 10% fetal bovine serum (FBS), 100 U/mL penicillin and 100 μg/mL streptomycin. Cells were cultured in a humidified incubator at 37°C with 5% CO_2_. AtT20 cells were obtained from the American Type Culture Collection (AtT20: CCL-89).

### RNA isolation

AtT20 cells grown to 70% confluence in regular medium in 24-multiwell plates were incubated either without or with DEX at appropriate concentrations in DMEM supplemented with 1% resin and charcoal-treated (stripped) FBS media [28,29] and cultured for 24 hrs. In the overexpression experiments, each expression vector was transfected for 24 hrs before treatment with DEX. The cells were then lysed and their total RNAs were isolated using ISOGEN (NIPPON GENE, Toyama, Japan) according to the manufacturer’s instructions. The RNA was quantified by a Nano drop 2000 (Thermo Scientific, Waltham, MA). The final preparation of total RNA was free of DNA and Proteins, and had a 260/280 ratio 1.6–1.9.

### Quantitative real-time PCR

Quantitative real-time polymerase chain reaction (qPCR) was performed using KAPA SYBR FAST Universal 2x qPCR Master Mix (KAPA Biosystems, Woburn, MA). One microgram from total RNA was converted to reverse transcription (RT) reaction using PrimeScript Reverse transcriptase (Takara Bio, Shiga, Japan) with the oligo-dT primer and random hexamer according to the manufacturer’s instructions. Reverse transcription mixtures were subjected to qPCR with KAPA SYBR FAST Universal 2x qPCR Master Mix (KAPA Biosystems) for primers of *Pomc*, *NeuroD1*, *Pan1* (*E47*), *Rb*, and *GAPDH* using a CFX connect Real Time PCR thermal cycler (Bio-Rad, Hercules, CA). The following primer sequences were used: mouse *Pomc* (forward, 5’-CAGTGCCAGGACCTCACC-3’; reverse, 5’-CAGCGAGAGGTCGAGTTTG-3’), mouse *NeuroD1* (forward, 5’-ACGCAGAAGGCAAGGTGTCC-3’; reverse, 5’-TTGGTCATGTTTCCACTTCC-3’), mouse *Pan1* (*E47*) (forward, 5’-GAATGCCTATGCCACCTTTG-3’; reverse, 5’-GAACCTTCCGGACCTTCTTC-3’), mouse *Rb* (forward, 5’-ATGGAATCCCTTGCATGGCT-3’; reverse, 5’-AGGACAAGCAGGTTCAAGGT-3’), mouse *Tpit* (forward, 5’-GCCAGCATGTGACCTACTCTCACT-3; reverse, 5’-AGTCCAGCTGTCAGGTCCCGAGAA-3’), mouse *Pitx1* (forward, 5-CGGTGTGGACCAACCTCACTGAA-3; reverse, 5’-GAGTTGCACGTGTCCCGGTAGA-3’) [27], mouse *Nur77* (forward, 5’-GCACAGCTTGGGTGTTGATG-3’; reverse, 5’-CAGACGTGACAGGCAGCTG-3’) [31], mouse *Nurr1* (forward, 5’-TCAGAGCCCACGTCGATT-3’; reverse, 5’-TAGTCAGGGTTTGCCTGGAA-3’) [32], mouse *GAPDH* (forward, 5’-ACAGTCCATGCCATCACTGCC-3’; reverse, 5’-GCCTGCTTCACCACCTTCTTG-3’), rat *Pomc* (forward, 5’-CCTCACCACGGAAAGCA-3’; reverse, 5’-TCAAGGGCTGTTCATCTCC-3’) [33] and rat *GAPDH* (forward, 5’-AAACCCATCACCATCTTCCA-3’; reverse, 5’-GTGGTTCACACCCATCACAA-3’) [34]. Reactions were incubated at 95°C for 1 min and then amplified using temperature parameters of 95°C for 15 sec; 60°C for 10 sec; 72°C for 20 sec. Amplifications were carried out for 45 cycles, followed by a 3 min extension at 72°C. After amplification, a melting-curve analysis was performed from 60°C to 95°C with a heating rate of 0.5°C/10 sec and continuous fluorescence acquisition. The signals of the samples of interest were then quantified from the standard curve, and all obtained data were normalized by mouse *GAPDH*. To confirm the amplification specificity, the PCR products from each primer pair with SYBR green were subjected to a melting curve analysis. Results are expressed as percentages of each control.

### Transient transfection and luciferase assay

AtT20 cells grown to 60–70% confluence in regular medium in 24-multiwell plates were transiently transfected with 300 ng of each reporter plasmid and 100 or 150 ng of β-gal control plasmid using Lipofectamine LTX and Plus reagent (Invitrogen) for 24 hrs according to the manufacturer’s instructions. In the overexpression experiments, different concentrations of each expression vector (200 ng and 300 ng), 135 ng of reporter plasmid and 65 ng of β-gal control plasmid were also transfected. The media were changed to DMEM supplemented with 1% stripped FBS, and the cells were treated without or with DEX (100 nM) and cultured for 24 hrs. These cells were then washed with 1xPBS and the cell extracts were prepared using Glo Lysis Buffer (Promega). Luciferase activity was measured using Bright-Glo reagents (Promega) and β-galactosidase activity was simultaneously measured. Data were normalized by β-galactosidase activity.

### Western blot analysis

When AtT20 cells were grown to 90–100% confluence in 6 well plates, they were exposed to DEX and maintained at different time courses (30 min, 1 hr, 3 hrs, 6 hrs, 9 hrs and 24 hrs) in DMEM supplemented with 1% stripped FBS. The cells were then harvested and lysed with TNE buffer [20 mM Tris-HCl, 137 mM NaCl, 2 mM EDTA, 1% NP-40, Protease Inhibitor Cocktail Set III (Calbiochem)]. This was followed by centrifuging at 13000 rpm for 10 min at 4°C to collect the supernatants. Thereafter, 10 μg of extracted protein were electrophoresed on a SDS–polyacrylamide gel and transferred onto polyvinylidene difluoride (PVDF) membrane. For the detection of NeuroD, the membrane was blocked with 1% BSA for 30 min and probed with primary rabbit monoclonal antibody for NeuroD (D3562, Cell Signalling Technology, Danvers, MA) (diluted at 1:500) for overnight at 4°C and was thereafter incubated with anti-rabbit IgG, horseradish peroxidase (HRP) linked whole antibody from donkey (NA934V, GE Healthcare, Little Chalfont, UK) (1:5000) for 1 h at room temperature. For the detection of actin, the membrane was blocked with 1% BSA for 30 min and probed with primary antibody for actin (sc-1616, Santa Cruz Biotechnology, Dallas, TX) (diluted at 1:500) for overnight at 4°C and was thereafter incubated with polyclonal rabbit anti-goat immunoglobulins/HRP (ab97120, Dako, Glostrup, Denmark) (1:5000) for 1 h at room temperature. The membranes were thereafter washed and visualized using ECL (Thermo Scientific) and Luminata forte for Actin and NeuroD1, respectively. Densitometric analyses of the membranes were performed using Image J.

### Chromatin immunoprecipitation (ChIP) assay

ChIP assay was conducted using NeuroD antibody (D3562, Cell Signalling Technology), normal rabbit IgG (SC-2027, Santa Cruz Biotechnology), and primers of mouse *Pomc* promoter containing the E-box region. Briefly, AtT20 cells were cross-linked with 0.5% formaldehyde for 10 min at 37°C and then glycine (final concentration 0.125 M) was added to stop the cross linking. Cells were washed twice with cold 1xPBS. Then, 1 ml cold 1xPBS was added and cells were scrapped into a 1.5 ml eppendorf tube and centrifuged at 1000g for 5 min at 4°C. The cell pellet was resuspended with 150 μl SDS lysis buffer [SDS lysis buffer: 1% SDS, 10 mM EDTA, 50 mM Tris-HCl (pH 8.1)] and kept on ice for 10 min. The cell lysate was sonicated to shear the DNA to fragment lengths of between 100 bp and 500 bp by using a handy sonic (Model UR-20 P) for 20 sec on and 60 sec off, 9 times at level 7. The sonicated extract was diluted 10 fold with dilution buffer [ChIP Dilution buffer: 0.01% SDS, 1.1% Triton X-100, 2 mM EDTA, 20mM Tris-HCl (pH 8.1), 167 mM NaCl]. Aliquotes of the lysate were incubated overnight with 2.0 μg of antibody at 4°C with rotation. The antibody-protein complex was precipitated by blocked protein G dynabeads and the pelleted beads were washed with wash buffers (Once with Low Salt Buffer, High Salt Buffer, LiCl Buffer and twice with TE Buffer). Immune complexes were eluted in elution buffer (1% SDS, 10 mM DTT, 0.1 M NaHCO_3_) and reverse cross linked with 5 M NaCl, then incubated for more than 6 hrs or overnight at 65°C. Following Proteinase K (Wako, Osaka, Japan) treatment, DNA fragments were then purified with a Qiagen DNA Extraction kit according to supplied protocol. Immunoprecipitated DNA was analyzed by qPCR using KAPA SYBR FAST Universal 2x qPCR Master Mix (KAPA Biosystems) together with 1% of the input chromatin. Specific primer pairs were designed to amplify the E-box region of mouse the *Pomc* gene promoter for qPCR. E-box primer **(**Forward primer, 5’- TACCTCCAAATGCCAGGAAG-3’; Reverse primer, 5’- GTTAGCACAGACCCGCTGA-3’).

### Primary culture of anterior pituitary cells

All animal experiments were approved by the Institutional Animal Experiment Committee of Jichi Medical University (Number:16052), and in accordance with the Institutional Regulation for Animal Experiments and Fundamental Guidelines for Proper Conduct of Animal Experiment and Related Activities in Academic Research Institutions under the jurisdiction of the Japanese Ministry of Education, Culture, Sports, Science and Technology. All treatments were performed under deep anesthesia and all efforts were made to minimize suffering. Rats were sacrificed by exsanguination from the right atrium under deep pentobarbital anesthesia (40 mg/kg body weight, i.p.) and then perfused with Hanks’ balanced salt solution in order to obtain anterior pituitary cells for primary culture.

Male Wistar rats were purchased from Japan SLC (Shizuoka, Japan). Rats were housed in a temperature-controlled room (22 ± 1°C) with a 12-hour light/12-hour dark cycle and illumination from 0700 h to 1900 h, and were given ad libitum access to conventional food and water. Anterior pituitary cells of rats aged 10–12 weeks were dispersed as described previously [35]. The cells were suspended in M199 medium (Life Technologies, Carlsbad, CA, USA) with 10% fetal bovine serum (FBS) and 1% penicillin-streptomycin. The cells (3 × 10^5^ cells) were seeded in 24-well plates and then incubated at 37°C in a humidified atmosphere of 5% CO_2_ and 95% air for 3 days. The culture medium was changed with M199 medium with 10% stripped FBS and then incubated for 1 hr. Media were changed, dexamethasone was then added to media at a concentration of 100 nM, 1 μM and the cells were incubated for 1 hr, 3 hrs, 6 hrs, or 24 hrs.

### Statistical analysis

All data are presented as means ± standard errors of means (SEM). Statistical analyses were performed with one way ANOVA followed by post hoc Tukey test and Paired Sample t test. *P*<0.05 was considered statistically significant.

## Results

### Effects of DEX on *Pomc* mRNA expression/*Pomc* promoter activity in AtT20 cells

**Fig 1:**
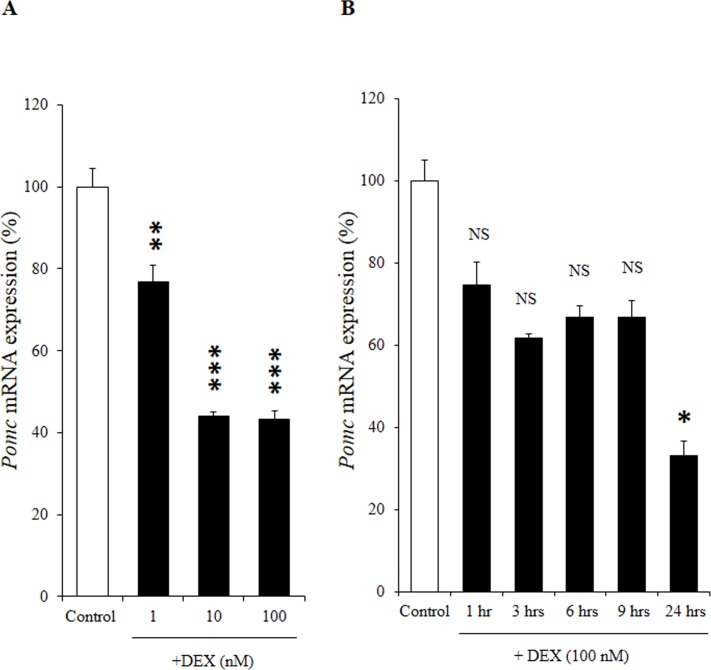
Effects of DEX on *Pomc* mRNA expression in AtT20 cells. (A) Dose-dependent effect of DEX on *Pomc* mRNA expression. AtT20 cells were treated with DEX (1 nM, 10 nM, or 100 nM) or 0.1% ethanol (vehicle control) for 24 hrs. Results are expressed as percentages of control (100%). Data represents mean ± SEM (n = 4). ***P<0.001 vs control. This experiment was repeated three times with separate batches of cell preparation with consistent results. (B) Time-dependent effect of DEX on *Pomc* mRNA expression. AtT20 cells were treated with 100 nM DEX for 1 hr, 3 hrs, 6 hrs, 9 hrs, or 24 hrs. Vehicle control: 0.1% ethanol. Results are expressed as percentages of control (100%). Each point represents mean ± SEM (n = 4). NS denotes “Not Significant.” ***P<0.001 vs control. This experiment was also repeated three times with separate batches of cell preparation with consistent results.

**Fig 2:**
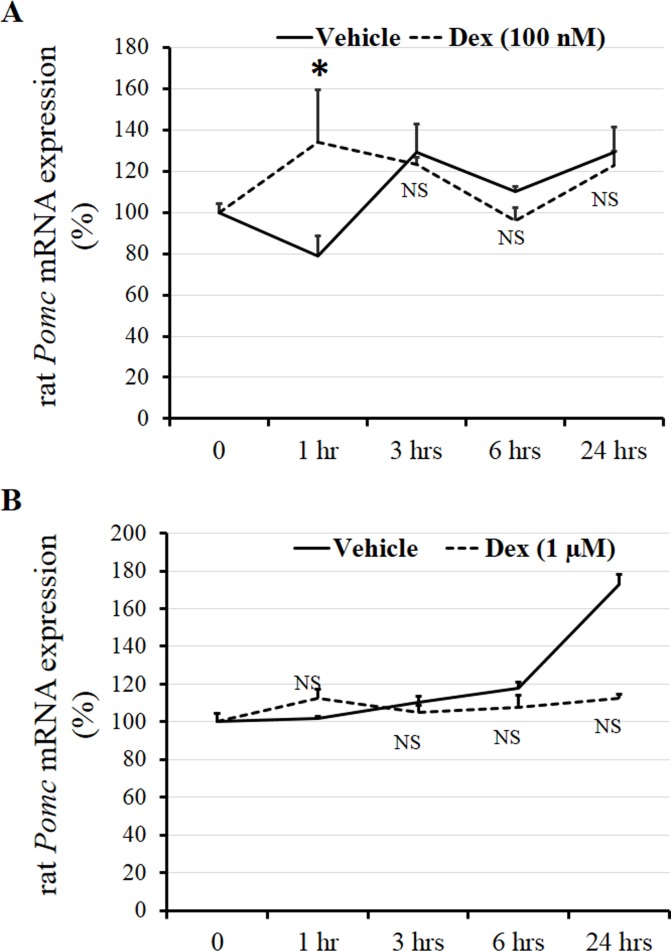
Effects of DEX on *Pomc* mRNA expression in primary culture cells of rat anterior pituitary. (A) Time-dependent effect of DEX on rat *Pomc* mRNA expression. Untreated rat anterior pituitary primary culture cells were expressed as percentages of control (100%). The cells were treated with 100 nM DEX or 0.1% ethanol (Vehicle control) for 1 hr, 3 hrs, 6 hrs, or 24 hrs. Each point represents mean ± SEM (n = 4). *P<0.05 vs control. NS denotes “Not Significant.” This experiment was repeated two times with separate batches of cell preparation with consistent results. (B) Time-dependent effect of DEX on rat *Pomc* mRNA expression. Untreated rat anterior pituitary primary culture cells were expressed as percentages of control (100%). The cells were treated with 1 μM DEX or 0.1% ethanol (Vehicle control) for 1 hr, 3 hrs, 6 hrs, or 24 hrs. Each point represents mean ± SEM (n = 4). NS denotes “Not Significant.”

**Fig 3:**
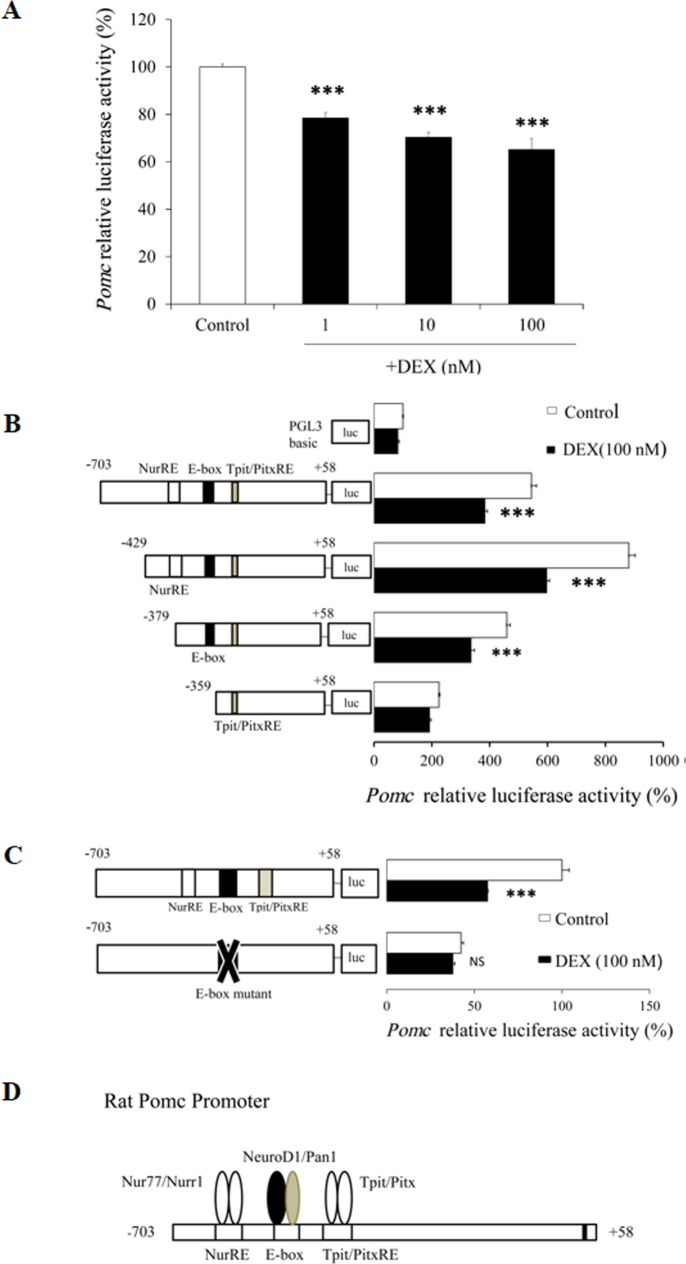
Effects of DEX on *Pomc* promoter activity in AtT20 cells. (A) Effect of DEX on full-length *Pomc* promoter activity. AtT20 cells transiently transfected with 300 ng full-length r*Pomc*-Luc (-703/+58-luc) and 100 ng pCMV-β-gal were treated with DEX (1 nM, 10 nM, or 100 nM) or 0.1% ethanol (vehicle control) for 24 hrs before the luciferase assay. Results are expressed as percentages of control (100%). Data represents mean ± SEM (n = 4). ***P<0.001 vs control. Three independent experiments were performed with consistent results. (B) Effect of DEX on *Pomc* promoter deletion mutants. AtT20 cells transiently transfected with 300 ng r*Pomc*-Luc (-703/+58-luc) or each deletion mutant reporter plasmid (-429/+58-Luc, -379/+58-Luc, or -359/+58-Luc) and 100 ng pCMV-β-gal were incubated in the presence (100 nM) or absence of DEX for 24 hrs before the luciferase assay. Results are expressed as percentages of each control (100% in pGL3-Basic). Each point represents mean ± SEM (n = 4). ***P<0.001 vs control. This experiment was also repeated three times with separate batches of cell preparation with consistent results. (C) Effect of DEX on *Pomc* promoter activity using E-box mutant. AtT20 cells transiently transfected with 300 ng rP*omc*-Luc (-703/+58-luc) or E-box mutant of full-length *Pomc* promoter (r*Pomc*-Luc-Ebox_Neuro_-Mut) and 100 ng pCMV-β-gal were incubated in the presence (100 nM) or absence of DEX for 24 hrs before the luciferase assay. Results are expressed as percentages of each control (100% in rP*omc*-Luc). Each point represents mean ± SEM (n = 4). NS denotes “Not Significant.” ***P<0.001 vs control. Two independent experiments were performed with consistent results. (D) Schematic representation of responsive elements in *Pomc* promoter and transcription factors which bind to these responsive elements.

We first examined the effect of DEX on *Pomc* mRNA expression at different concentrations in AtT20 cells. After treatment of the AtT20 cells with several concentrations (1 nM, 10 nM, and 100 nM) of DEX, *Pomc* mRNA expression was dose-dependently decreased (Fig 1A). We next investigated the effects of DEX on *Pomc* mRNA expression with different incubation durations in AtT20 cells. After treatment of the AtT20 cells with DEX (100 nM) for different incubation durations (1 hr, 3 hrs, 6 hrs, 9 hrs, and 24 hrs), the *Pomc* mRNA expression was time-dependently decreased, significantly at 24 hrs (Fig 1B). These data indicate that DEX decreased *Pomc* mRNA expression in both dose- and time-dependent manners. In contrast, when we treated primary culture cells of rat anterior pituitary with DEX (100 nM) and DEX (1 μM) for different incubation durations (1 hr, 3 hrs, 6 hrs, and 24 hrs), *Pomc* mRNA expression was not suppressed significantly comparing to vehicle control (Fig 2A and 2B) consistent with the previous conference report [36] though Dex (100 nM) caused a small but significant transient increase in POMC transcription after 1 hour of treatment (Fig 2A). We next examined the effects of DEX on the *Pomc* promoter activity using AtT20 cells. In this experiment, we used the full-length (-703/+58) *Pomc* promoter in its 5’-flanking region with different concentrations of DEX. As shown in Fig 3A, DEX significantly decreased the *Pomc* promoter activity in a dose-dependent manner. These data indicate that DEX negatively regulates *Pomc* transcription.

### Role of E-box of *Pomc* promoter in the DEX-mediated suppression in AtT20 cells

In order to understand the molecular mechanisms of the DEX-mediated *Pomc* transcription regulation, we examined the promoter activity of a series of *Pomc* 5’-flanking region deletion mutants. The DEX-mediated transcription suppression of *Pomc* promoter activity was observed in constructs from the full-length (-703/+58) to -379/+58, but not in -359/+58 (Fig 3B). The luciferase activity of pGL3-Basic Vector was not affected by DEX (Fig 3B). *Pomc* constructs from -703/+58 to -429/+58 contained both the Nur response element (NurRE) and E-box, whereas the -379/+58 construct contained the E-box but not NurRE. Since both -429/+58 and -379/+58 constructs exhibited similar DEX-induced inhibition (Fig 3B), it is plausible that not only NurRE but also E-box may play an important role in the DEX-mediated transcription suppression. In order to further clarify the role of E-box in the transcription suppression, we next examined the effect of DEX on the E-box point mutant. As shown in Fig 3C, the transcription of the E-box point mutant was not affected by DEX, indicating the significance of E-box in the DEX-mediated transcription suppression. Fig 3D shows the structure of the rat *Pomc* promoter. Since Nur77/Nurr1 [37] is known to bind to NurRE, and NeuroD1 [7] is known to bind to E-box, not only Nur77/Nurr1 but also NeuroD1 may be involved in the DEX-mediated suppression of the *Pomc* promoter activity.

### Effects of DEX on mRNA expression of *NeuroD1*, *Pan1*, and *Rb* in AtT20 cells

**Fig 4:**
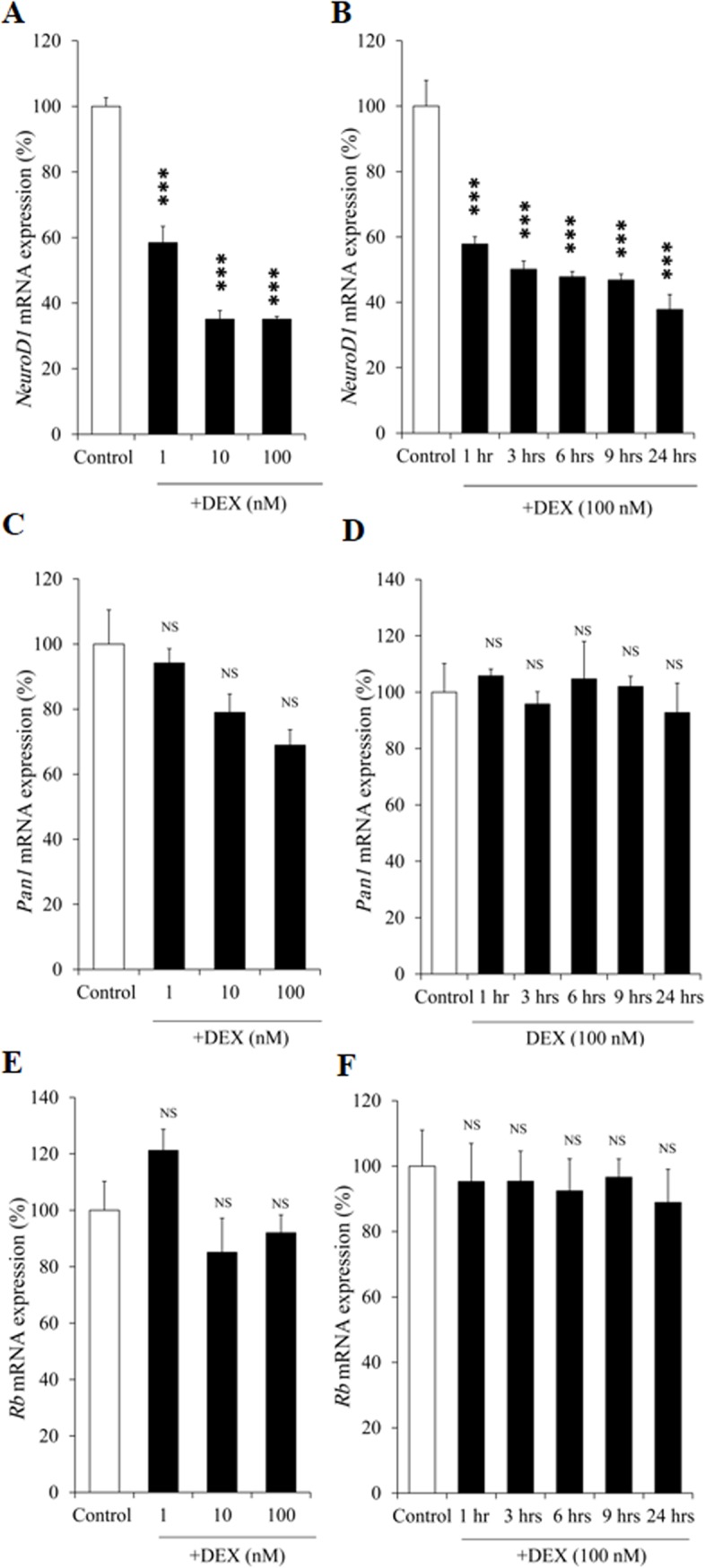
Effects of DEX on the mRNA expression of *NeuroD1*, *Pan1* (*E47*), and *Rb* in AtT20 cells. (A) Dose-dependent effect of DEX on *NeuroD1* mRNA expression. AtT20 cells were treated with DEX (1 nM, 10 nM, or 100 nM) or 0.1% ethanol (vehicle control) for 24 hrs. Results are expressed as percentages of control (100%). Each point represents mean ± SEM (n = 4). ***P<0.001 vs control. This experiment was also repeated three times with separate batches of cell preparation with consistent results. (B) Time-dependent effect of DEX on *NeuroD1* mRNA expression. AtT20 cells were treated with DEX (100 nM) for 1 hr, 3 hrs, 6 hrs, 9 hrs, or 24 hrs. Vehicle control: 0.1% ethanol. Results are expressed as percentages of control (100%). Each point represents mean ± SEM (n = 4). ***P<0.001 vs control. (C) Dose-dependent effect of DEX on *Pan1* (*E47*) mRNA expression. AtT20 cells were treated with DEX (1 nM, 10 nM, or 100 nM) or 0.1% ethanol (vehicle control) for 24 hrs. Results are expressed as percentages of control (100%). NS denotes “Not Significant.” This experiment was also repeated three times with separate batches of cell preparation with consistent results. (D) Time-dependent effect of DEX on *Pan1* (*E47*) mRNA expression. AtT20 cells were treated with DEX (100 nM) for 1 hr, 3 hrs, 6 hrs, 9 hrs, or 24 hrs. Vehicle control: 0.1% ethanol. Results are expressed as percentages of each control (100%). NS denotes “Not Significant.” (E) Dose-dependent effect of DEX on *Rb* mRNA expression. AtT20 cells were treated with DEX (1 nM, 10 nM, or 100 nM) or 0.1% ethanol (vehicle control) for 24 hrs. Results are expressed as percentages of control (100%). NS denotes “Not Significant.” (F) Time-dependent effect of DEX on *Rb* mRNA expression. AtT20 cells were treated with DEX (100 nM) for 1 hr, 3 hrs, 6 hrs, 9 hrs, or 24 hrs. Vehicle control: 0.1% ethanol. Results are expressed as percentages of control (100%). NS denotes “Not Significant.”

We next examined the effects of DEX on *NeuroD1* mRNA expression in AtT20 cells. As shown in Fig 4A and 4B, DEX decreased the *NeuroD1* mRNA expression in dose- and time-dependent manners. These data indicate that the DEX-mediated *Pomc* transcription suppression may be mediated via the decrease of *NeuroD1* mRNA expression. We next examined the effect of DEX on *Pan1* (*E47*) mRNA expression, since Pan1 is known to be a heterodimeric partner of NeuroD1 [7]. As shown in Fig 4C and 4D, DEX did not affect the *Pan1* mRNA expression, probably due to its ubiquitous expression. Additionally, since Retinoblastoma protein (Rb) has recently been demonstrated to be a co-activator of NeuroD1 on the *Pomc* [38], we also examined the effect of DEX on *Rb* mRNA expression. As shown in Fig 4E and 4F, *Rb* mRNA expression was not affected by DEX.

### Effects of DEX on mRNA expression of *Tpit*, *Pitx1*, *Nur77*, and *Nurr1* in AtT20 cells

**Fig 5:**
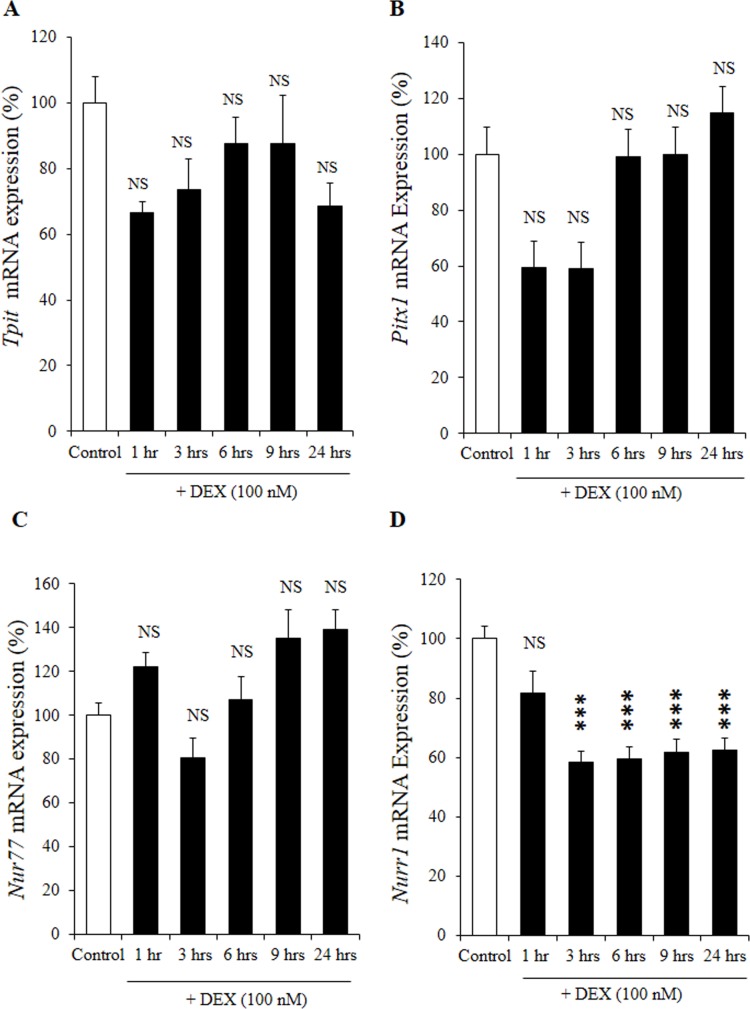
Effects of DEX on the mRNA expression of *Tpit*, *Pitx1*, *Nur77* and *Nurr1* in AtT20 cells. (A) Time-dependent effect of DEX on *Tpit* mRNA expression. AtT20 cells were treated with DEX (100 nM) for 1 hr, 3 hrs, 6 hrs, 9 hrs, or 24 hrs. Vehicle control: 0.1% ethanol. Results are expressed as percentages of control (100%). Each point represents mean ± SEM (n = 4). NS denotes “Not Significant.” Two independent experiments were performed with consistent results. (B) Time-dependent effect of DEX on *Pitx1* mRNA expression. AtT20 cells were treated with DEX (100 nM) for 1 hr, 3 hrs, 6 hrs, 9 hrs, or 24 hrs. Vehicle control: 0.1% ethanol. Results are expressed as percentages of each control (100%). NS denotes “Not Significant.” Two independent experiments were performed with consistent results. (C) Time-dependent effect of DEX on *Nur77* mRNA expression. AtT20 cells were treated with DEX (100 nM) for 1 hr, 3 hrs, 6 hrs, 9 hrs, or 24 hrs. Vehicle control: 0.1% ethanol. Results are expressed as percentages of control (100%). NS denotes “Not Significant.” Two independent experiments were performed with consistent results. (D) Time-dependent effect of DEX on *Nurr1* mRNA expression. AtT20 cells were treated with DEX (100 nM) for 1 hr, 3 hrs, 6 hrs, 9 hrs, or 24 hrs. Vehicle control: 0.1% ethanol. Results are expressed as percentages of control (100%). Each point represents mean ± SEM (n = 4). NS denotes “Not Significant.” ***P<0.001 vs control. Two independent experiments were performed with consistent results.

We also examined the effects of DEX on *Tpit* and *Pitx1* mRNA expression in AtT20 cells. As shown in Fig 5A and 5B, DEX did not decrease *Tpit* and *Pitx1* mRNA expression in time-dependent manners. This is consistent to the previous report by Murakami et al. [27] which demonstrated that DEX did not suppress mRNA expression of *Tpit* and *Pitx1*. These data indicate that the DEX-mediated *Pomc* transcription suppression may not be mediated via the decrease of *Tpit* and *Pitx1* mRNA expression. Moreover, while DEX did not suppress *Nur77* mRNA expression (Fig 5C), it significantly decreased *Nurr1* mRNA expression in a time-dependent manner (Fig 5D). These data indicate that the DEX-mediated *Pomc* transcription suppression may also be mediated via the decrease of *Nurr1* mRNA expression, although further studies are needed to clarify its mechanism.

### Effects of DEX on *NeuroD1* promoter activity in AtT20 cells

**Fig 6:**
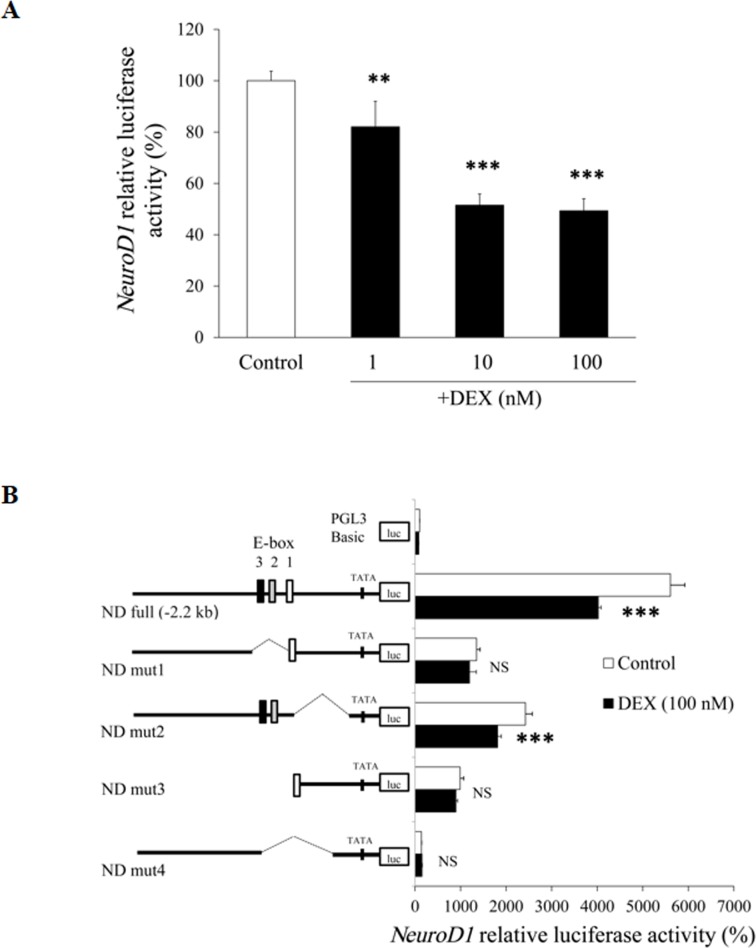
Effects of DEX on *NeuroD1* promoter activity in AtT20 cells. (A) Effect of DEX on full-length *NeuroD1* promoter activity. AtT20 cells transiently transfected with 300 ng m*NeuroD1*-Luc (ND full: -2.2-kb/+150) and 150 ng pRSV-β-gal were treated with DEX (1 nM, 10 nM, or 100 nM) or 0.1% ethanol (vehicle control) for 24 hrs before the luciferase assay. Results are expressed as percentages of control (100%). Each point represents mean ± SEM (n = 4). **P<0.01 and ***P<0.001 vs control. (B) Effect of DEX on *NeuroD1* promoter deletion mutants. AtT20 cells transiently transfected with 300 ng ND full, ND mut1, ND mut2, ND mut3, or ND mut4 and 150 ng pRSV-β-gal were incubated in the presence (100 nM) or absence of DEX for 24 hrs before the luciferase assay. Results are expressed as percentages of each control (100% in pGL3-Basic). Each point represents mean ± SEM (n = 4). ***P<0.001 vs control. NS denotes “Not Significant.”

In order to elucidate the mechanisms of the DEX-mediated decrease of the *NeuroD1* mRNA expression, we next examined the effect of DEX on the *NeuroD1* promoter activity using AtT20 cells. When the full-length (-2.2-kb/+150) *NeuroD1* promoter in its 5’-flanking region was incubated with several concentrations of DEX for 24 hrs, DEX significantly suppressed the *NeuroD1* promoter activity in a dose-dependent manner (Fig 6A). These data indicate that DEX also negatively regulates the *NeuroD1* transcription.

### Role of E-box(es) of *NeuroD1* promoter in the DEX-mediated suppression in AtT20 cells

In order to identify the element(s) responsible for the DEX-mediated suppression of *NeuroD1* transcription, we examined the promoter activity of the *NeuroD1* 5’-flanking region deletion mutants. As shown in Fig 6B, there exist 3 types of E-box (denoted as E-box1, 2 and 3) on the *NeuroD1* promoter. The DEX-mediated transcription suppression of the *NeuroD1* promoter activity was observed in the full-length *NeuroD1* promoter (ND full) and in ND mut2 that lacks E-box1, but not in ND mut1/ND mut3 that lack E-box2/3 or in ND mut4 that lacks E-box1/2/3. Taken together, the E-box2 and/or E-box3 may be important for the DEX-mediated negative expression of *NeuroD1*.

### Effects of DEX on NeuroD1 protein expression/NeuroD1-*Pomc* E-box interaction in AtT20 cells

**Fig 7:**
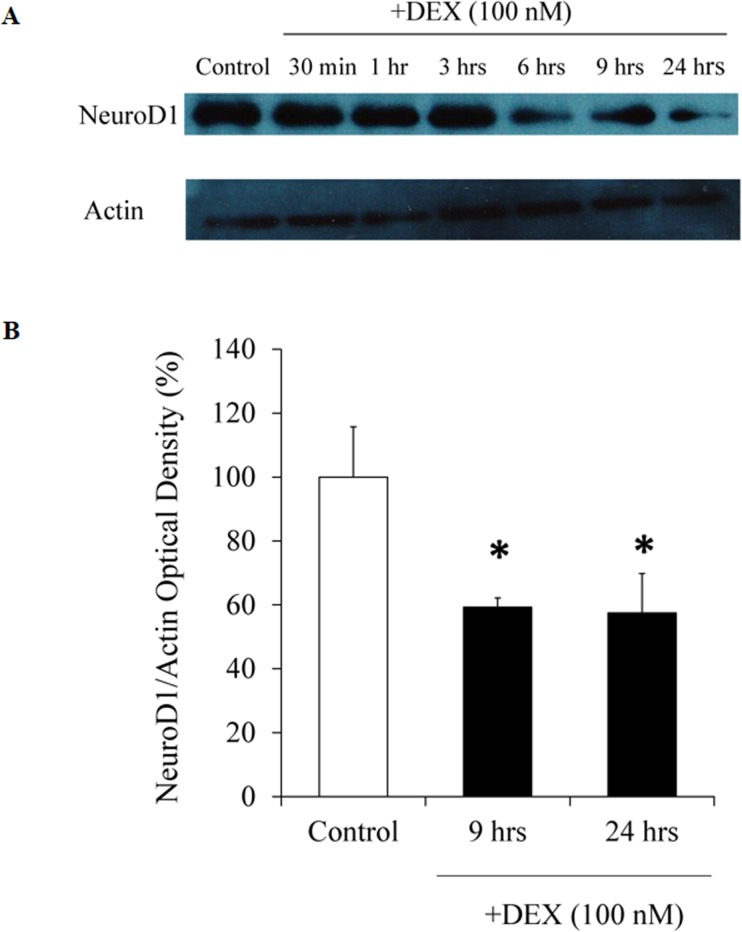
Effects of DEX on NeuroD1 protein expression in AtT20 cells. (A) Time-dependent effect of DEX on NeuroD1 and actin protein expression. AtT20 cells were treated with DEX (100 nM) for 1 hr, 3 hrs, 6 hrs, 9 hrs, or 24 hrs before the Western blot analyses. Vehicle control: 0.1% ethanol. In (B), AtT20 cells were treated with DEX (100 nM) for 9 hrs or 24 hrs before the Western blot analyses. Vehicle control: 0.1% ethanol. Optical density (OD) of NeuroD1 was normalized by OD of actin. Results are expressed as percentages of control (100%) Each point represents mean ± SEM (n = 3). **P<0.01vs control. Two independent experiments were performed with consistent results.

**Fig 8:**
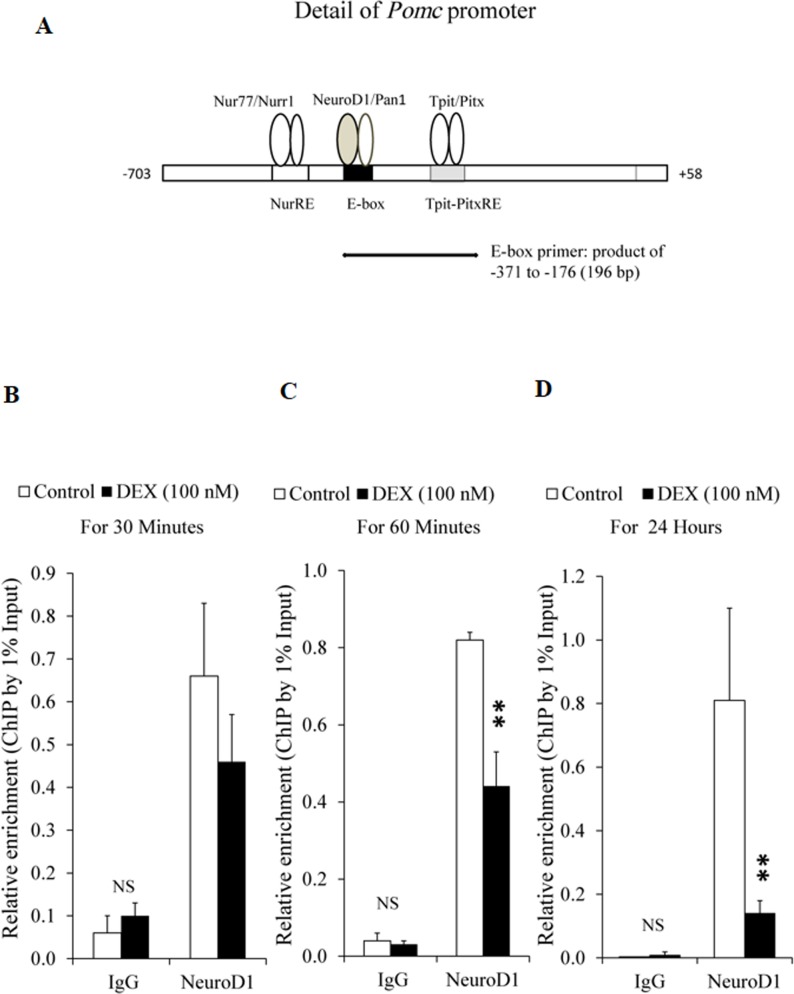
Effects of DEX on interaction between NeuroD1 and E-box on *Pomc* promoter in AtT20 cells. (A) Effect of of DEX on interaction between NeuroD1 and E-box on *Pomc* promoter examined by ChIP assay using E-box primer. ChIP assay was performed using digested chromatin extracted from the cells cultured in the presence (100 nM) or absence (control) of DEX for 30 min (B), 60 min (C), or 24 hrs (D). Chromatin fragments were immunoprecipitated either by normal rabbit IgG (negative control) or NeuroD1 antibody. Purified DNA was analyzed by qPCR using primers specific for E-box containing sequence on *Pomc* promoter. The expected size of E-box is 196 bp. Few qPCR products observed in the input samples were detected in the immunoprecipitation using normal IgG. Immunoprecipitated DNA was quantified by qPCR and normalized to the values obtained after amplification of unprecipitated 1% input DNA. Each point represents mean ± SEM (n = 3). **P<0.01, significantly different from the level of control group. These experiments were repeated three times with separate batches of cell preparation with consistent results.

We next examined the effects of DEX on NeuroD1 protein expression. As shown in Fig 7A and 7B, DEX suppressed the protein expression of NeuroD1 after more than 6 hrs, but not by 3 hrs. Since NeuroD1 is known to bind to the E-box of the *Pomc* promoter [7], we further examined the effects of DEX on the interaction of NeuroD1 and E-box of the *Pomc* promoter by ChIP assay using primers encompassing E-box (Fig 8A). We then examined the effect of DEX on the interaction between NeuroD1 and E-box of the *Pomc* promoter using NeuroD antibody by ChIP assay. Although treatment with DEX for 30 min did not affect the interaction between NeuroD1 and E-box (Fig 8B), DEX treatment for 60 min significantly suppressed their interaction (Fig 8C). Moreover, DEX treatment for 24 hrs further suppressed the interaction between NeuroD1 and E-box (Fig 8D). When control IgG was used, DEX treatment did not affect their interaction at any time point (Fig 8B, 8C, and 8D). Taken together, it is speculated that DEX not only suppress the interaction between NeuroD1 and E-box through the decrease of NeuroD1 protein expression in the long run (24 hrs), but also through the inhibition of their binding in the short run (60 min).

### Effects of NeuroD1 overexpression on the DEX-mediated suppression of *Pomc* mRNA expression/*Pomc* promoter activity in AtT20 cells

**Fig 9:**
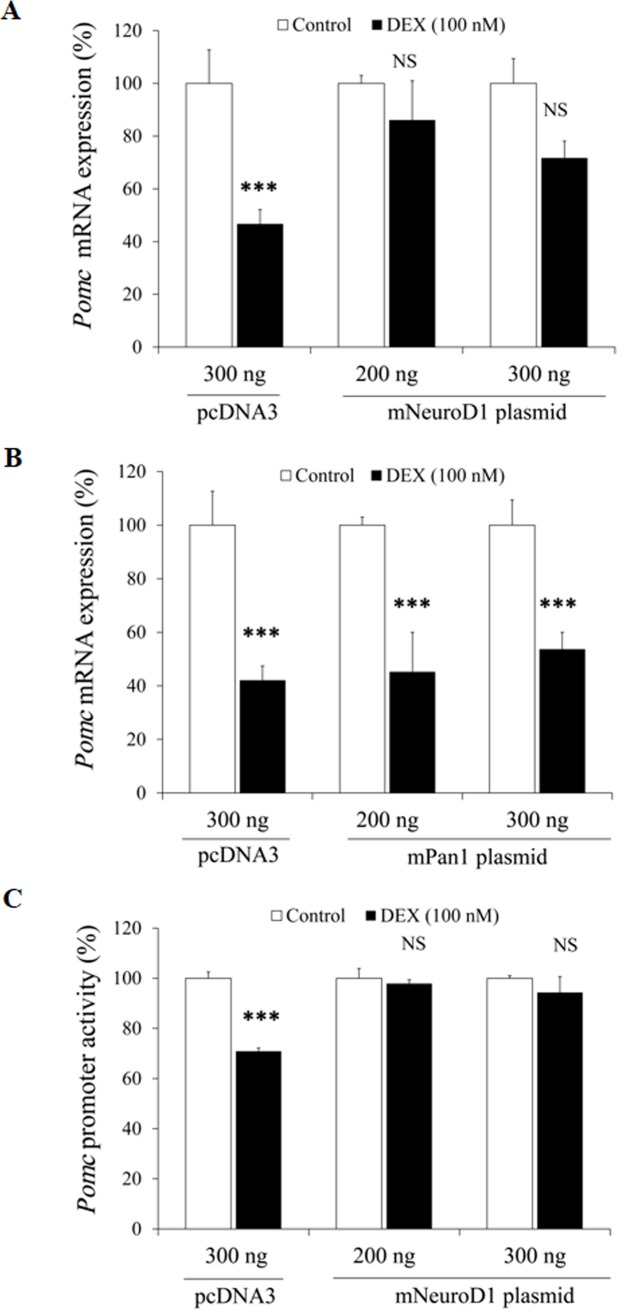
Effects of NeuroD1 overexpression on *Pomc* mRNA expression and *Pomc* promoter activity in AtT20 cells. (A) Effect of NeuroD1 overexpression on the DEX-mediated *Pomc* mRNA decrease. AtT20 cells were transiently transfected with pcDNA3 and NeuroD1 plasmid (volume adjusted to 300 ng each with pcDNA3 empty vector) and incubated either in the presence (100 nM) or absence (control) of DEX for 24 hrs. Results are expressed as percentages of each control (100%). Each point represents mean ± SEM (n = 4). ***P<0.001 vs control. NS denotes “Not Significant.” Two independent experiments were performed with consistent results. (B) Effect of Pan1 overexpression on the DEX-mediated *Pomc* mRNA decrease. AtT20 cells were transiently transfected with pcDNA3 and Pan1 plasmid (volume adjusted to 300 ng each with pcDNA3, empty vector) and incubated either in the presence (100 nM) or absence (control) of DEX for 24 hrs. Results are expressed as percentages of each control (100%). Each point represents mean ± SEM (n = 4). ***P<0.001 vs control. Two independent experiments were performed with consistent results. (C) Effect of overexpression of NeuroD1 on the DEX-mediated suppression of *Pomc* promoter activity. AtT20 cells were transiently transfected with pcDNA3 and NeuroD1 plasmid (volume adjusted to 300 ng each with pcDNA3 empty vector), 135 ng r*Pomc*-Luc, and 65 ng pRSV-β-gal were incubated either in the presence (100 nM) or absence (control) of DEX for 24 hrs before the luciferase assay. Results are expressed as percentages of each control (100%). Each point represents mean ± SEM (n = 4). ***P<0.001 vs control. NS denotes “Not Significant.” Two independent experiments were performed with consistent results.

We next induced the overexpression of NeuroD1 in order to examine the involvement of NeuroD1 in the DEX-mediated suppression. As shown in Fig 9A, overexpression of NeuroD1 rescued the DEX-mediated suppression of *Pomc* mRNA expression, while that of control plasmid (pcDNA3) did not. We next induced the overexpression of Pan1. As shown in Fig 9B, Pan1 overexpression did not rescue the DEX-mediated suppression of *Pomc* mRNA expression. NeuroD1 overexpression also rescued the DEX-mediated suppression of *Pomc* promoter activity (Fig 9C). These data also indicate the involvement of NeuroD1 in the DEX-mediated *Pomc* suppression.

## Discussion

The effects of Gc on *Pomc* transcription in rat pituitary was first demonstrated in 1984 in a study [19] showing that both DEX and corticosterone suppressed *Pomc* transcription in the anterior lobe, but not in the intermediate lobe. Additionally, DEX was shown to be more potent than corticosterone in supressing *Pomc* transcription [19], consistent with its long biological half-life as well as relative anti-inflammatory potency [39]. However, the mechanism of the negative regulation of *Pomc* transcription by Gc is still unclear in many points.

Two rat *Pomc* promoter fragments including either -4.5-kb or -706-bp 5'-flanking region were previously examined for the Gc-mediated inhibition, and it was observed that these two promoter fragments were almost equally suppressed by Gc [18]. Therefore, the *Pomc* promoter 5'-flanking region including -706-bp seems to be sufficient for obtaining the Gc-mediated *Pomc* transcription suppression. We therefore investigated the mechanisms of DEX on the *Pomc* promoter activity using the -703/+58 construct and its deletion mutants (Fig 3A and 3B). Several transcription factor binding sites have been shown to exist on the *Pomc* promoter region [40], and synergistic interactions among multiple regulatory elements are required for *Pomc* transcription in the pituitary [41]. Among them, two Nur binding sites have been identified on the *Pomc* promoter. The proximal binding sequence termed Nur77-binding response element (NBRE) (-69/-63) is known to be bound by the Nur77 monomer [40,42], and the distal Nur response element (NurRE), constituted of two inverted NBRE related sites (-404/-397 and -390/-383), is known to be bound by the Nur77 homodimer or Nur77/Nurr1 heterodimer; and the (distal) NurRE responds to Nur77 much stronger than the (proximal) NBRE [37,40,43]. Moreover, Tpit/Pitx-responsive elements (-316/-309 and -302/-297) [40,44] and NF-κB-responsive element (-151/-142) [40,45] are also known to be involved in *Pomc* regulation. Additionally, E-box (-377/-370) [7,40,46], to which NeuroD1 (β2) binds as a heterodimer with Pan1 (E47) for transactivation, has been known to play an important role in *Pomc* transcription regulation [7]. Moreover, NeuroD1 is known to regulate various genes in endocrine, enteroendocrine, and neuroendocrine cells such as insulin 1 [47,48], glucokinase (*GK*) [49], secretin [50], inositol 1,4,5-triphosphate receptor 1 *(IP3R1)* [51], and early B-cell factor 3 *(Ebf3)* [52].

We here have first observed the dose- and time-dependent effect of DEX on the suppression of *NeuroD*1 mRNA expression in AtT20 cells (Fig 4A and 4B). Regarding *Tpit* and *Pitx1* mRNA expression, Gc has been reported to have no effect [27]. Although Pan1 is well known as the heterodimeric partner of NeuroD1, DEX did not affect the mRNA expression of *Pan1* (Fig 4C and 4D), probably due to its ubiquitousness [9]. Recently, Rb has been reported to function as a coactivator of NeuroD1 for *Pomc* transcription in AtT20 cells [38]. However, *Rb* mRNA expression was not affected by DEX (Fig 4E and 4F). It is therefore indicated that the DEX-mediated inhibition of *NeuroD1* mRNA expression may be involved in the Gc-mediated *Pomc* transcription suppression. Interestingly, the DEX-mediated *Pomc* transcription suppression was ameliorated by the overexpression of NeuroD1 (Fig 9A and 9C), but not by that of its heterodimeric partner Pan1 (Fig 9B). These data further suggest the functional significance of NeuroD1 in the transcription suppression of *Pomc*.

Since DEX also suppressed *NeuroD1* promoter activity (Fig 6A), it is likely that the DEX-mediated inhibition of *NeuroD1* mRNA expression is mediated at the gene transcription level. Analyses of the *NeuroD1* promoter region using deletion mutants revealed that E-box2 and/or E-box3 may be involved in the Gc-mediated transcriptional suppression in AtT20 cells (Fig 6B). Among E-box1, 2 and 3 in the 5’-flanking region of *NeuroD1*, E-box1 and 3 have been reported to play an important role in islet-specific *NeuroD1* expression [53]. Moreover, the sequence of E-box3 in the *NeuroD1* promoter is identical to that of E-box in the *Pomc* promoter, which determines its corticotroph specific expression [7]. Further studies are needed to clarify which E-box in the *NeuroD1* promoter determines its corticotroph specific expression.

We also examined the effect of DEX on the NeuroD1 protein expression by Western blot analyses. DEX significantly inhibited the NeuroD1 protein expression after more than 9 hrs (Fig 7A and 7B). This time course may be differ among cell lines since DEX inhibited NeuroD1 protein expression after more than 2 hrs in insulin-secreting pancreatic HIT-T15 cells [54]. Moreover, we have first demonstrated the inhibitory effects of DEX on the interaction of NeuroD1 and E-box in the *Pomc* promoter by ChIP assay using AtT20 cells (Fig 8). Although the DEX-mediated decrease of NeuroD1 protein expression could be observed after more than 6 hrs (Fig 7A), DEX significantly inhibited the interaction between NeuroD1 and E-box after 60 min. Since 60 min incubation of DEX did not affect the NeuroD1 protein expression (Fig 7A), it is speculated that DEX may inhibit the binding of NeuroD1 to E-box independently of the NeuroD1 protein decrease, most likely via its epigenetic effect.

*Pomc* gene transcription is regulated by coordination of several transcription factors including NeuroD1. Although NeuroD1 is an important transcription factor of *Pomc* gene, NeuroD1 and Tpit/Pitx transcription factors have synergistic effect on *Pomc* gene regulation [9]. Moreover, the combination of Pitx1, Tpit and NeuroD1 has been suggested to be enough to specify the genetic program of corticotroph cells compared to other anterior pituitary lineages, while NeuroD1 is not expressed in melanotroph cells of the intermediate lobe of pituitary at any time during development or in adults [55]. Since we have also observed the DEX-mediated suppression of *Nurr1* mRNA expression (Fig 5D), the interaction of NeuroD1 and Nurr1 in the Gc-mediated negative regulation of *Pomc* is further needed to be elucidated.

**Fig 10:**
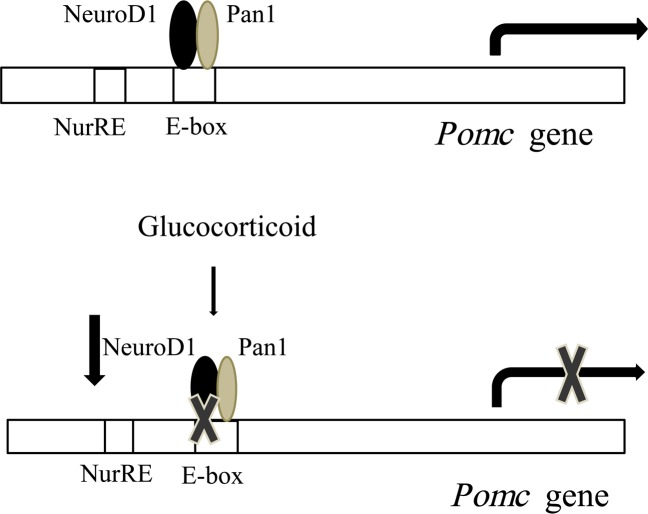
Involvement of NeuroD1 in the Gc-mediated transcription suppression of *Pomc*.

Our observation is summarized in Fig 10. NeuroD1 binds to E-box on the *Pomc* promoter and enhances *Pomc* gene expression [7]. However, when Gc is added, it decreases *NeuroD1* transcription, mRNA expression, and protein expression, resulting in the decrease of NeuroD1 binding to E-box on the *Pomc* promoter to suppress *Pomc* transcription. It can therefore be concluded that NeuroD1 may play an important role in the negative regulation of *Pomc* expression in AtT20 cells by Gcs.
